# Preferences of healthcare workers for provider payment systems in The Gambia’s National Health Insurance Scheme

**DOI:** 10.1186/s12913-023-09885-8

**Published:** 2023-08-11

**Authors:** Hassan Njie, Patrick G. C. Ilboudo, Unni Gopinathan, Lumbwe Chola, Knut Reidar Wangen

**Affiliations:** 1 Department of Community Medicine and Global Health, University of Oslo, Postboks 1130, Blindern, Oslo, 0318 Norway; 2 African Population and Health Research Center, Nairobi, Kenya; 3 Cluster for Global Health, Division for Health Services, Norwegian Institute of Public Health, Oslo, Norway; 4 Department of Health Management and Health Economics, University of Oslo, Oslo, Norway

**Keywords:** Healthcare financing, Universal health care, Strategic purchasing, Provider payment systems, Provider performance

## Abstract

**Background:**

The Government of The Gambia introduced a national health insurance scheme (NHIS) in 2021 to promote universal health coverage (UHC). Provider payment systems (PPS) are strategic purchasing arrangements that can enhance provider performance, accountability, and efficiency in the NHIS. This study assessed healthcare workers’ (HCWs’) preferences for PPS across major service areas in the NHIS.

**Methods:**

A facility-based cross-sectional study was conducted using a probability proportionate to size sampling technique to select an appropriate sample size. Health care workers were presented with options for PPS to choose from across major service areas. Descriptive statistics explored HCW socio-demographic and health service characteristics. Multinomial logistic regressions were used to assess the association between these characteristics and choices of PPS.

**Results:**

The majority of HCW did not have insurance coverage, but more than 60% of them were willing to join and pay for the NHIS. Gender, professional cadre, facility level, and region influenced HCW’s preference for PPS across the major service areas. The preferred PPS varied among HCW depending on the service area, with capitation being the least preferred PPS across all service areas.

**Conclusion:**

The National Health Insurance Authority (NHIA) needs to consider HCW’s preference for PPS and factors that influence their preferences when choosing various payment systems. Strategic purchasing decisions should consider the incentives these payment systems may create to align incentives to guide provider behaviour towards UHC. The findings of this study can inform policy and decision-makers on the right mix of PPS to spur provider performance and value for money in The Gambia’s NHIS.

## Background

Improving health system performance and making progress towards Universal Health Coverage (UHC) are among the most pressing global health goals, particularly in low- and middle-income countries (LMICs). Studies have shown that a well-functioning health system, which includes a sufficient and competent health workforce, is essential for ensuring equitable access to quality health services [1, 2]. A well-functioning health workforce is necessary for achieving UHC because they can provide quality health services that are responsive to the needs of the population [3]. Similarly, a strong health workforce can help to reduce health inequities and promote universal access to health services [3–5].

The Gambia’s Ministry of Finance and Economic Affairs (MoFEA) introduced programme-based budgeting in 2016 with a view to improving health sector priorities, allocative efficiency, and accountability for results. However, since its introduction, input-based payment by budget line- items dominates the health financing landscape with strong features of passive purchasing. For example, public sector HCW receive monthly salaries without linking them to provider performance and accountability.

Countries that have made progress towards UHC use strategic purchasing levers to allocate resources efficiently, create deliberative incentives to enhance quality, access and equitable services as well as ensure provider autonomy and accountability [6, 7]. Strategic purchasing is a key component of health financing that involves the efficient and effective allocation of financial resources to improve health system performance and health outcomes [6]. Health financing, consisting of the three core functions of revenue raising, pooling and purchasing [8], plays a crucial role in strengthening health workforce. Strategic purchasing is about purchasing agencies such as ministries of health (MoH), health insurance agencies and other purchasers making active, evidence-based decisions about what services to purchase, from which providers, how these services are paid for and at what price [9, 10].

A core feature of strategic purchasing are PPS, which refer to methods in which purchasers transfer funds to individual HCW or provider institutions to provide agreed services to the population [11]. Provider payment systems can create strong incentives that influence provider behaviour and invariably, the efficiency, equity and quality outcomes of NHIS [12]. PPS is a critical component of any NHIS and is an essential factor in achieving UHC [13, 14]. In addition, the type of payment system utilized in a NHIS can also help control health care costs by creating incentives to providers to deliver care in the most efficient way possible [15]. Strategic purchasing decisions should consider incentives various PPS create and how these influence HCW behaviour and accountability. This is especially important in The Gambia where HCW have embarked on a series of industrial strikes demanding better salaries and incentives [16, 17].

Studies have consistently demonstrated that both financial and non-financial incentives can influence HCW behaviour and contribute to positive patient outcomes [18–20]. For example, the implementation of performance-based financing to incentivize HCWs under the Maternal and Child Nutrition and Health Results Project (MCNHRP) in The Gambia showed a higher quality of care (QoC) score in targeted facilities (71.3%) compared to non-targeted facilities (36.8%) [21]. HCWs in the targeted regions also reported higher levels of satisfaction due to the incentives they received in addition to their monthly salaries [22, 23]. However, some researchers have argued that financial incentives alone may not be sufficient to improve patient outcomes due to inconclusive or weak evidence of their impact on service quality [24–26].

The Gambian government established the NHIS in 2021, as a crucial step towards achieving UHC. The NHIS implementation in The Gambia is being overseen by the National Health Insurance Authority (NHIA), which is actively exploring various provider payments systems to establish a framework for incentivizing healthcare providers while ensuring accountability and value for money to enhance the efficiency of the scheme.

This study investigates the preferences of HCWs for payment systems and incentives to inform strategic purchasing decisions by the NHIA.

## Methods

### Study setting

**Fig. 1:**
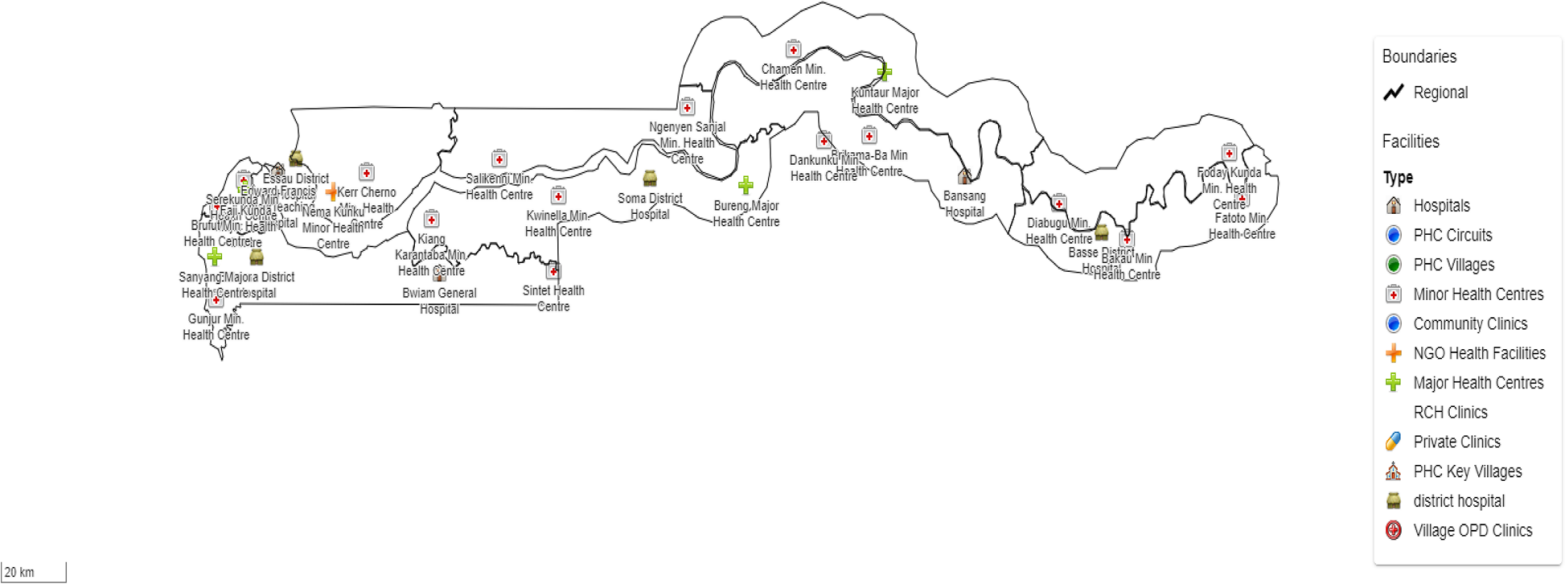
Distribution of study facilities

This study was conducted in The Gambia between August and September 2020, utilizing a nationwide facility-based cross-sectional survey design (see Fig. 1). The MoH has demarcated the country into seven health regions, including Western 1 Region (W1R), Western 2 Region (W2R), North Bank West Region (NBWR), North Bank East Region (NBER), Lower River Region (LRR), Central River Region (CRR), and Upper River Region (URR), as part of its efforts to decentralize health service delivery.

### Sampling approach

#### Sample size estimation

The focus of our study is individual healthcare workers (HCWs) working in public health facilities. We originally intended to include hospital administrators, as they make important decisions within their respective hospitals. However, due to a low response rate among hospital administrators and the fact that the majority of our participants were employed by the MoH rather than hospitals, we ultimately excluded hospital administrators from our sample. To determine the appropriate sample size for our study, we used Raosoft’s online sample size calculator with the following conservative assumptions: there are 5,000 public healthcare workers in The Gambia, a 95% confidence interval, a 5% margin of error, and a 50% response distribution. The estimated sample size was 357 participants, but we increased it by 60% to 576 participants due to concerns about potential low response rates due to COVID-19 restrictions.

#### Sampling technique

We utilized a two-stage sampling technique to select participants from their respective facilities. In the first stage, we selected 57 public health facilities using a combination of systematic sampling and probability proportionate to size technique, stratified by region and tier to ensure representativeness. We sampled 60% of facilities from each stratum, resulting in 32 public health facilities being eligible for selection in the final sample. Sampling 60% of the total health facilities allowed us to achieve our targeted sample size of 576 participants, the unit of analysis in our study. In the second stage, we used a systematic sampling technique to determine the sampling interval for each health facility. To ensure gender and sub-cadre representativeness, healthcare workers were stratified by gender, cadre and qualifications based on each facility’s sampling interval. We adapted the multiple indicator cluster survey 6 (MICS6) systematic random selection template, which has been validated and used in previous surveys and studies such as the 2018 MICS, 2019 Gambia demographic and health survey, and 2020 Gambia integrated household survey [27–30]. The health facility’s weekly or monthly duty roster served as the sampling frame for our study.

Our study included HCWs working in public facilities who were on duty at the time of data collection and gave written consent. We excluded non-Gambian HCWs, as well as those working in private for-profit, non-profit, and faith-based facilities. HCWs in the private sector were excluded due to some management’s reluctance to share human resource data needed for probability proportional to size sampling. Due to the COVID-19 pandemic, HCWs reassigned to COVID-19 duties, on home isolation or quarantine due to infections, were also excluded. Additionally, HCWs on annual, casual, or sick leave, as well as HCWs on duty but not found on site during data collection, were excluded from our study.

### Study instrument

We collected relevant information from the respondents using a self-administered questionnaire. To ensure an objective response without ambiguities, we presented simple and precise definitions of the different provider payment systems as described in the WHO UHC technical brief [31]. To avoid disrupting health service delivery, respondents were strongly encouraged to complete the questionnaire in their own time. We designed the instrument based on a previously validated tool [13] and content validated it with researchers who have expertise in PPS. Furthermore, we pre-tested the tool among 20 HCWs in a public health facility, which was subsequently excluded from the final survey.

### Statistical analysis

The outcome variable of our study was the preference for PPS, which we grouped into the six most common PPS used for health insurance schemes in LMICs. These PPS were global budget, line-item budget, fee-for-service, capitation, case-based (DRG), and per diem. Our explanatory variables included socio-demographic characteristics of HCWs, such as gender and type of cadre, as well as health facility characteristics, including level of facility and region. We analyzed the following main service areas: primary outpatient services, hospital outpatient services, inpatient services (hospitalization), and referral services. We categorized the service areas based on the lead author’s knowledge of the healthcare delivery system in The Gambia and a similar study [13].

Considering the limited PPS studies in Sub-Saharan Africa [13, 32] and the unique three-tier health care delivery system in The Gambia, we hypothesized that female HCWs would be less likely to prefer case-based payment for all service areas, while physicians would prefer fee-for-service as the payment system for all service areas. Additionally, we hypothesized that HCWs in hospitals would prefer case-based payment (DRG) for hospitalization, and those in rural areas would prefer capitation as the payment system for primary and hospital outpatient services, as shown in other studies [33, 34].

We described the demographic and socio-economic characteristics of the respondents, as well as other relevant factors. Furthermore, we used multinomial logistic regression models to estimate HCWs’ preferences for the PPS for different service areas, including primary outpatient services, hospital outpatient services, inpatient services (hospitalization), and referral services. We used global budget as the reference PPS category in the models. We performed all statistical analyses using Stata/SE 17.0.

### Patient and public involvement

This study did not include patients, and the public was not involved in the conceptualization and finalization of the questionnaire. However, some researchers with healthcare backgrounds who worked in clinical settings in the past supported the pre-test of the questionnaire by providing suggestions for more clarity. Those who were involved in this process were not included in the study. The authors plan to organize a dissemination forum in The Gambia at policy, facility, and community levels to share the key research findings.

## Results

### Demographic and socio-economic characteristics of HCWs

Table 1 shows the demographic and socio-economic characteristics of the respondents. The majority of the respondents (76.7%) work within urban centres, with female HCW constituting 53.3% of the study population. More than two-thirds of respondents were between 19–40 years of age, and 66.4% were married.

**Table 1:** Demographic and socio-economic characteristics of HCWs

	All (n, %)	Urban (n, %)	Rural (n, %)
Local Government Area	576 (100.0)	442 (76.7)	134 (23.3)
Gender			
Female	303 (53.3)	222 (39.0)	81 (14.2)
Male	266 (46.7)	213 (37.4)	53 (9.3)
Age (in years)			
19–29	192 (44.3)	137 (41.8)	55 (52.4)
30–40	168 (38.8)	135 (41.2)	33 (31.4)
41–51	64 (14.8)	50 (15.2)	14 (13.3)
> 51	9 (2.1)	6 (1.8)	3 (2.9)
Marital status			
Never married	173 (30.1)	124 (21.6)	49 (8.5)
Married	381 (66.4)	296 (51.6)	85 (14.8)
Living together, divorced, separated, widow	20 (3.5)	20 (3.5)	0 (0.0)
Level of education			
Certificate	224 (39.1)	155 (27.1)	69 (12.0)
Diploma, Higher National Diploma	186 (32.5)	139 (24.3)	47 (8.2)
Degree (Bachelor’s, Master’s, PhD)	163 (28.5)	145 (25.3)	18 (3.1)
Monthly income (in GMD)			
< 500–9,999	404 (72.7)	289 (52.0)	115 (20.7)
10,000–19,999	110 (19.8)	95 (17.1)	15 (2.7)
> 19,999	42 (7.6)	42 (7.6)	0 (0.0)

United States Dollar 1 (US$) = Gambian Dalasi (GMD) 48.13; Exchange rate (July–August 2020); Local Government Area = distribution of facilities based on region

The highest level of education attained that was most common among the HCWs was certificate (39.1%) and fewer HCWs in rural areas had university degrees (3.1%) compared to their urban counterparts (25.3%). More than two-thirds of respondents (72.7%) had monthly income of less than GMD 10,000 (US$ 207.77).

### Other characteristics of HCWs

Table 2 shows that nurses and midwives formed the largest proportion of the study population (70.4%). About 5.5% of HCW indicated that they were not licensed to practice. More than a third (40.0%) had work experience ranging from less than one to three years. The majority of HCW indicated that they had no private health insurance coverage (96.9%). When asked whether they were willing to join and pay for NHIS, 82.1% agreed. A small proportion of HCW (15.8%) stated that the health insurance scheme should reimburse individual HCW for health services rendered to the population instead of health facilities. About 87.1% stated that there should be a gatekeeper policy in NHIS.

**Table 2:** Other descriptive characteristics of HCWs

	All (n, %)	Urban (n, %)	Rural (n, %)
Profession			
Physician	46 (8.0)	45 (7.8)	1 (0.2)
Nurse, midwife	406 (70.5)	303 (52.6)	103 (17.9)
Other cadre	124 (21.5)	94 (16.3)	30 (5.2)
Licensed to practice			
Yes	483 (94.5)	368 (72.0)	115 (22.5)
No	28 (5.5)	23 (4.5)	5 (1.0)
Work experience (in years)			
< 1–3	229 (40.0)	171 (29.9)	58 (10.1)
4–9	175 (30.6)	137 (24.0)	38 (6.6)
> 9	168 (29.4)	131 (22.9)	37 (6.5)
Private insurance cover			
Yes	16 (3.1)	14 (2.7)	2 (0.4)
No	504 (96.9)	388 (74.6)	116 (22.3)
Willingness to join and pay for NHIS			
Yes	437 (82.1)	332 (62.4)	105 (19.7)
No	95 (17.9)	77 (14.5)	18 (3.4)
NHIS to reimburse health facility instead of individual HCW			
Yes	470 (84.2)	355 (63.6)	115 (20.6)
No	88 (15.8)	71 (12.7)	17 (3.1)
Gate keeper in NHIS			
Yes	488 (87.1)	369 (65.9)	119 (21.3)
No	72 (12.9)	58 (10.4)	14 (2.5)

### HCW preference for payment system associated with primary outpatient services

Table 3 shows HCW working in district hospital or major health centres are 50% less likely to choose line-item budgeting (RRR = 0.5; 95% CI = (0.3,1.0) and fee-for-service (RRR = 0.5; 95% CI = (0.3, 1.0)) compared to those working in hospitals. Furthermore, working in an urban area is associated with 60% less likelihood (RRR = 0.4; 95% CI = (0.2, 0.7)) of choosing case-based payment relative to rural-based HCWs.

**Table 3:** Results of multinomial logistic regression for primary outpatient services

	Line-item budget		Capitation		Case-based (DRG)		Fee-for-service		Per-diem	
	RRR (95% CI)	*P*	RRR (95% CI)	*P*	RRR (95% CI)	*P*	RRR (95% CI)	*P*	RRR (95% CI)	*P*
**Gender**										
Male (ref)										
Female	1.6 (0.9, 2.7)	0.08	1.0 (0.5, 1.9)	0.94	1.5 (0.8, 2.7)	0.20	1.4 (0.9, 2.2)	0.19	1.4 (0.6, 3.0)	0.45
**Professional cadre**										
Other cadre (ref)										
Physician	0.8 (0.2, 2.7)	0.71	0.7 (0.2, 2.7)	0.64	2.8 (0.8, 9.7)	0.10	0.7 (0.2, 2.0)	0.46	0.3 (0.1, 2.9)	0.31
Nurse/midwife	1.6 (0.8, 3.1)	0.17	0.8 (0.4, 1.6)	0.50	2.2 (1.0, 5.1)	0.06	1.5 (0.8, 2.6)	0.20	1.1 (0.4, 2.8)	0.86
**Facility level**										
Hospital (ref)										
Minor H.C	1.1 (0.6, 2.2)	0.67	1.3 (0.6, 2.8)	0.52	1.0 (0.5, 2.3)	0.97	1.3 (0.7, 2.3)	0.36	0.9 (0.3, 2.5)	0.86
Dist. Hos./ M.H.C	0.5 (0.3, 1.0)	**0.05**	0.4 (0.2, 1.0)	0.06	0.9 (0.4, 1.9)	0.82	0.5 (0.3, 1.0)	**0.05**	0.6 (0.2, 1.6)	0.32
**Region**										
Rural (ref)										
Urban	0.6 0.3, 1.1)	0.10	0.7 (0.3, 1.6)	0.43	0.4 (0.2, 0.7)	**0.01**	0.8 (0.4, 1.4)	0.40	0.7 (0.3, 1.9)	0.53

*Global budget* Base reference, *Ref* Reference, *RRR* Relative risk ratio, *CI* Confidence interval, *P P*-value, significant = 0.05, *Dist. Hos.* District hospital, *M.H.C* Major health centre

### HCW preference for payment system associated with hospital outpatient services

Table 4 shows that being a physician is associated with higher likelihood of choosing line-item budget by almost four times (RRR = 3.9; 95% CI = (1.2, 12.0) and case-based payment by six times (RRR = 6.0; 95% CI = (1.9, 18.7) than other cadres. Nurses or midwives are twice as likely to choose case-based payment compared to other cadres (RRR = 2.0; 95% CI = (1.0, 3.8). Working in a hospital is negatively associated with choosing case-based payment (RRR = 0.3; 95% CI = (0.2, 0.6) than working in district hospital or major health centre. HCWs in urban areas are strongly associated with line-item budget as payment system for hospital outpatient services (RRR = 2.1; 95% CI = (1.0, 4.4) relative to HCW in rural areas.

**Table 4:** Results of multinomial logistic regression for hospital outpatient services

	Line-item budget		Capitation		Case-based (DRG)		Fee-for-service		Per-diem	
	RRR (95% CI)	*P*	RRR (95% CI)	*P*	RRR (95% CI)	*P*	RRR (95% CI)	*P*	RRR (95% CI)	*P*
**Gender**										
Male (ref)										
Female	1.3 (0.7, 2.3)	0.39	0.9 (0.4, 1.9)	0.78	0.8 (0.5, 1.3)	0.35	1.1 (0.7, 1.8)	0.65	0.6 (0.3, 1.2)	0.14
**Professional cadre**										
Other cadre (ref)										
Physician	3.9 (1.2, 12.0)	**0.02**	1.4 (0.3, 6.9)	0.68	6.0 (1.9, 18.7)	**< 0.01**	1.1 (0.4, 3.6)	0.83	1.6 (0.3, 7.7)	0.57
Nurse/midwife	1.6 (0.8, 3.2)	0.21	1.1 (0.4, 2.6)	0.91	2.0 (1.0,3.8)	**0.05**	1.2 (0.7, 2.1)	0.52	1.0 (0.4, 2.3)	0.94
**Facility level**										
Minor H.C (ref)										
Dist. Hos. / M.H.C	1.4 (0.6, 3.0)	0.42	1.0 (0.4, 3.1)	0.94	0.6 (0.3, 1.1)	0.09	0.8 (0.4, 1.4)	0.40	0.9 (0.4, 2.5)	0.90
Hospital	0.9 (0.4, 2.0)	0.87	1.6 (0.6, 4.3)	0.36	0.3 (0.2, 0.6)	**< 0.01**	1.0 (0.5, 1.8)	0.96	0.8 (0.3, 2.0)	0.66
**Region**										
Rural (ref)										
Urban	2.1 (1.0, 4.4)	**0.05**	0.8 (0.3, 1.8)	0.54	1.2 (0.7, 2.2)	0.54	0.8 (0.5, 1.5)	0.54	1.8 (0.7, 4.7)	0.26

*Global budget* Base reference, *Ref* Reference, *RRR* Relative risk ratio, *CI* Confidence interval, *P P*-value, significant = 0.05, *Dist. Hos.* District hospital, *M.H.C* Major health centre

### HCW preference for payment system associated with inpatient services (hospitalization)

Table 5 shows that being a female is negatively associated with per diem as preferred choice of payment for hospitalization by sixty percent (RRR = 0.4; 95% CI = (0.2, 0.8) relative to being a male. Similarly, working in a district hospital or major health centre is negatively associated with the choice of capitation by seventy percent (RRR = 0.3; 95% CI = (0.1, 0.9) compared to working in a hospital. Urban-based HCWs are significantly associated with a less likelihood of choosing capitation by seventy percent (RRR = 0.3; 95% CI = (0.1, 0.6) relative to rural-based dwellers.

**Table 5:** Results of multinomial logistic regression for inpatient services (hospitalization)

	Line-item budget		Capitation		Case-based (DRG)		Fee-for-service		Per-diem	
	RRR (95% CI)	*P*	RRR (95% CI)	*P*	RRR (95% CI)	*P*	RRR (95% CI)	*P*	RRR (95% CI)	*P*
**Gender**										
Male (ref)										
Female	1.4 (0.7, 2.8)	0.31	0.7 (0.3, 1.5)	0.35	0.8 (0.5, 1.3)	0.47	1.2 (0.7, 2.0)	0.57	0.4 (0.2, 0.8)	**0.01**
**Professional cadre**										
Other cadre (ref)										
Physician	0.4 (0.1, 1.9)	0.24	2.5 (0.6, 10.6)	0.21	1.4 (0.6, 3.7)	0.46	0.6 (0.2, 1.9)	0.36	1.0 (0.2, 4.4)	0.96
Nurse/midwife	0.8 (0.4, 1.7)	0.64	1.4 (0.6, 3.6)	0.47	1.3 (0.7, 2.3)	0.38	1.4 (0.7, 2.7)	0.30	1.3 (0.6, 2.9)	0.53
**Facility level**										
Minor H.C (ref)										
Dist. Hos. / M.H.C	0.4 (0.2, 1.0)	0.06	0.3 (0.1, 0.9)	**0.03**	0.6 (0.3, 1.1)	0.09	0.6 (0.3, 1.2)	0.17	0.7 (0.3, 1.5)	0.34
Hospital	0.6 (0.3, 1.2)	0.15	0.7 (0.3, 1.8)	0.46	0.7 (0.4, 1.2)	0.17	0.9 (0.4, 1.7)	0.68	0.5 (0.2, 1.1)	0.12
**Region**										
Rural (ref)										
Urban	0.8 (0.4, 1.7)	0.51	0.3 (0.1, 0.6)	**< 0.01**	1.0 (0.5, 1.8)	0.90	0.8 (0.4, 1.5)	0.45	1.0 (0.5, 2.3)	0.95

*Global budget* Base reference, *Ref* Reference, *RRR* Relative risk ratio, *CI* Confidence interval, *P P*-value, significant = 0.05, *Dist. Hos.* District hospital, *M.H.C* Major health centre

### HCW preference for payment system associated with referral services

Table 6 shows evidence for association between being a female HCW and a preference for fee-for-service as payment system for referrals 1.7 (RRR = 1.7; 95% CI = (1.1, 2.7) compared to being a male. Physicians are more than three times as strongly associated with choosing case-based as payment vehicle for referrals (RRR = 3.3; 95% CI = (1.1, 10.1) compared to other cadres. HCWs in district hospitals or major health centres are negatively associated with capitation (RRR = 0.4; 95% CI = (0.2, 0.9) and per-diem (RRR = 0.4; 95% CI = (0.2, 1.0) compared to HCWs in hospitals. Furthermore, HCWs in urban areas are associated with less likelihood to choose capitation and case-based by sixty percent (RRR = 0.4; 95% CI = (0.2, 0.8) than HCW in rural areas. In addition, they are negatively associated with per-diem as payment vehicle for referral services by fifty percent (RRR = 0.5; 95% CI = (0.2, 1.0). All these associations are significant, with the data providing support for rejecting the null hypothesis.

**Table 6:** Results of multinomial logistic regression for referral services

	Line-item-budget		Capitation		Case-based (DRG)		Fee-for-service		Per-diem	
	RRR (95% CI)	*P*	RRR (95% CI)	*P*	RRR (95% CI)	*P*	RRR (95% CI)	*P*	RRR (95% CI)	*P*
**Gender**										
Male (ref)										
Female	1.1 (0.6, 2.1)	0.76	1.0 (0.6, 1.8)	0.96	0.9 (0.5, 1.7)	0.86	1.7 (1.1, 2.7)	**0.03**	1.4 (0.7, 2.8)	0.30
**Professional cadre**										
Other cadre (ref)										
Physician	0.7 (0.2, 2.6)	0.56	0.4 (0.1, 1.6)	0.21	3.3 (1.1, 10.1)	**0.04**	1.2 (0.4, 3.3)	0.77	0.3 (0.1, 2.8)	0.29
Nurse/midwife	0.9 (0.4, 1.8)	0.70	0.6 (0.3, 1.2)	0.13	1.2 (0.5, 2.5)	0.72	1.5 (0.8, 2.7)	0.22	1.2 (0.5, 2.8)	0.66
**Facility level**										
Minor H.C (ref)										
Dist. Hos. / M.H.C	0.6 (0.2, 1.4)	0.24	0.4 (0.2, 0.9)	**0.03**	1.5 (0.7, 3.5)	0.31	0.7 (0.4, 1.3)	0.24	0.4 (0.2, 1.0)	**0.05**
Hospital	1.6 (0.8, 3.5)	0.21	1.3 (0.6, 2.5)	0.50	1.8 (0.8, 4.2)	0.16	0.9 (0.5, 1.7)	0.84	1.0 (0.4, 2.1)	0.92
**Region**										
Rural (ref)										
Urban	0.6 (0.3, 1.3)	0.19	0.4 (0.2, 0.8)	**0.01**	0.4 (0.2, 0.8)	**0.01**	1.1 (0.6, 2.0)	0.80	0.5 (0.2, 1.0)	**0.05**

*Global budget* Base reference, *Ref* Reference, *RRR* Relative risk ratio, *CI* Confidence interval, *P P*-value, significant = 0.05, *Dist. Hos.* District hospital, *M.H.C* Major health centre

## Discussion

Our study aimed to analyze the associations between HCW characteristics and their preference for PPS in major service areas. Our findings revealed strong associations between HCW gender, cadre, and their preference for PPS. Furthermore, we observed strong associations between health facility characteristics, including facility level and region, and HCW preference for PPS across major service areas.

Our study did not find any significant negative association between females and case-based payment. However, we observed a strong negative association between females and per-diem as a preferred payment system for hospitalization relative to males, which contradicts our initial hypothesis. This finding contrasts with other studies that have reported fee-for-service as being poorly rated compared to other payment systems [35]. It is worth noting that per-diem reimbursement for services provided under health insurance schemes is uncommon in LMICs. In The Gambia, per-diem reimbursement is mainly applicable to domestic and international travel, workshops, meetings, and training. Female HCWs’ low preference for per-diem as a payment system in the NHIS in The Gambia may be due to their perception of low per-diem rates in The Gambia compared to neighboring countries like Senegal.

Our findings also indicated a positive association between females and fee-for-service payment for referral services compared to males. This contrasts with studies conducted in Nigeria and Ghana, which found that HCWs least preferred fee-for-service reimbursement compared to other payment systems [13, 32]. We did not find any significant association between gender and payment systems for all other service areas. Contrary to our hypothesis, we observed high variation in physicians’ preference for fee-for-service, which contrasts with its popularity in many countries, including LMICs [36–38]. Our findings are consistent with studies conducted in NHIS-implemented countries in SSA, which reported that HCWs rated fee-for-service less favorably than other payment systems [13, 35]. The negative association between physicians and fee-for-service in our study could be attributed to Gambia’s open health system. In the public sector, doctors may operate clinics or work part-time in private health facilities, pharmacies, and drug stores. HCWs in the public sector receive monthly salaries via traditional line-item budgets, while major private clinics pay doctors fee-for-service. However, the fee-for-service in the private sector is unstructured, and the unit price is influenced by many factors, such as working on weekends, nights, or public holidays. Consequently, doctors’ incomes tend to increase when they work during these periods, making their income unpredictable. Some physicians may have experienced the unpredictable nature of fee-for-service in the private sector, which may have influenced their decision to prefer other payment systems.

Our questionnaire responses from physicians were compatible with a positive and significant association with line-item budgets or case-based payment for hospital outpatient services and case-based payment for referral services. Several contextual factors may explain these preferences. Firstly, in The Gambia, case-based payment is similar to monthly salaries paid via line-item budgets because HCWs receive a fixed amount per case, per month regardless of costs incurred [39]. These payment systems offer doctors predictability in monthly income, which contrasts with fee-for-service. Conversely, a study conducted in Kenya reported mixed results, where HCWs perceived both capitation and fee-for-service as good sources of revenue for health providers [34].

Our study found a negative association between HCWs in hospitals and case-based payment for hospitalization, contradicting our hypothesis. In some countries implementing NHIS in Sub-Saharan Africa (SSA), case-based payment or modified case-based payment systems such as Ghana’s DRG system are used to pay for services rendered during hospitalization. Moreover, numerous studies have documented that HCWs prefer payment systems that offer higher payment rates [10, 35, 40]. Given that hospitals provide more specialist services, including procedures that could generate higher revenue for both the institution and individuals, it is surprising that this was not the case in our study. A plausible explanation for our finding may be that HCWs in hospitals are risk-averse and therefore prefer payment systems that are more familiar and predictable.

Our study did not find any significant association between HCWs in rural areas and their preference for capitation as a payment system for primary and hospital outpatient services compared to urban-based HCWs. This finding contradicts our hypothesis, which was based on the fact that in rural Gambia, the MoH allocates each public health facility with a sub-population to serve depending on the location, level, and scope of the health facility. These sub-populations are referred to as catchment area populations (CAP). For example, all rural-based public health facilities, including hospitals, are part of the performance-based financing arrangements, whereby agreed services they provide to their respective CAP are remunerated following verifications. Each facility generates a costed quarterly business plan to procure medicines, supplies, equipment, and other needs of the facility with consideration to the health needs of the CAP. The remuneration that health facilities receive following verified submission of their business plan is similar to capitation, and as such, we expected that HCWs in rural areas would choose this payment method relative to others. Our findings suggests that other factors, besides performance-based financing arrangements, may influence healthcare worker (HCW) preferences for payment systems in rural areas. Future studies are needed to identify these factors and explore the reasons for the lack of a strong association between rural-based HCWs and their preference for capitation as a payment system.

Additionally, the NHIA should consider the context-specific factors that influence HCW preferences for payment systems. For example, the unpredictable nature of fee-for-service in the private sector may influence HCW preferences for other payment systems. Furthermore, the risk-averse nature of HCW in hospitals may lead them to prefer payment systems that are more familiar and predictable.

The selection of PPS should consider HCW preferences to enhance provider performance and accountability, while also aligning with UHC goals, including utilization relative to needs, financial protection, and equity [41]. Country-specific factors such as macroeconomic situation, fiscal space for health, and PPS utilization as a blended or standalone method should also be considered. For example, in Ghana, the National Health Insurance Authority (NHIA) customized Diagnosis-Related Group (DRG) payments as part of its cost containment strategies [42]. Therefore, periodic reviews of the chosen payment system should be conducted to assess the effects of the incentives on HCW performance and accountability, as well as their impact on health system priorities and goals [11].

Our decision to exclude non-Gambian HCW was based on our experience during the pre-test, which showed difficulties in determining their work permission and license to practice in The Gambia. Additionally, we excluded HCWs who were on COVID-19 duties, home isolation, or quarantine due to the regulations set by the government for COVID-19 prevention and control. It is worth noting that their exclusion did not impact our findings.

### Strengths and limitations

This nationally representative study has several strengths that enhance its robustness and reliability. First, the study design allowed for all public health facilities, except for basic facilities, to have an equal chance of being included, which improves the generalizability of the findings. Second, the use of an intra-strata sampling technique, such as probability proportional to size, provided equal representation for gender and cadres of healthcare workers, including those with different qualifications, such as registered nurse, state enrolled nurse, and community health nurse. However, the study also had some limitations that need to be considered when interpreting the findings. Firstly, the study only focused on public health facilities, and private facilities were excluded due to their reluctance to share human resource data for sampling. Although it is acknowledged that many private sector HCWs work in the public sector, it would have been beneficial to include private sector HCWs for a more comprehensive view. Secondly, the low response rate from hospital administrators meant that their preferences were not included in the study. This is a potential limitation, as hospital administrators may be engaged by the NHIA during selection of PPS and their preferences could have enriched the findings. Finally, despite our efforts to explain the different PPS to the participants by providing definitions on the questionnaire, the majority of the HCWs were not practically familiar with them, which may have limited their understanding of the implications of choosing different PPS.

Despite these limitations, this study provides valuable insights into the preferences of public sector HCWs regarding payment systems in the Gambia, which can inform the development and implementation of the NHIS. Future studies may benefit from including all HCWs and hospital administrators, for instance by applying interviews or qualitative methods, as well as exploring ways to enhance the understanding of different PPS among HCWs.

## Conclusions

Our study provides valuable insights into HCW preferences for payment systems in The Gambia, indicating the need for a blended approach suitable for different health services and providers. For example, case-based payment or bundled payment methods may be appropriate for hospitalization services, while capitation, and fee-for-service used for some priority services, may be appropriate for primary care services. In addition, performance- based financing may also be appropriate for primary care services particularly services provided at village health services level in The Gambia. As The Gambia prepares to implement the NHIS, our findings can guide policymakers at the MoH and NHIA in selecting the right mix of payment systems to support progress towards UHC. By involving HCW in the process and considering context-specific factors as reported in other studies, the NHIA can ensure that the chosen payment systems are politically and culturally acceptable, feasible, and sustainable for all stakeholders involved [13, 32].

## Data Availability

The dataset used and/or analyzed during the current study are available from the corresponding author on reasonable request.

## Abbreviations

NHIS: National Health Insurance Scheme
PPS: Provider payment systems
HCW: Health care workers
UHC: Universal health coverage
LMIC: Low- and middle- income countries
LIC: Low income countries
SDG: Sustainable development goals
HRH: Human resources for health
MCNHRP: Maternal and child nutrition and health results project
QoC: Quality of care
NHIA: National health insurance agency
W1 R: Western 1 Region
W2 R: Western 2 Region
NBWR: North Bank West Region
NBER: North Bank East Region
LRR: Lower River Region
CRR: Central River Region
URR: Upper River Region
MICS6: Multiple Indicator Cluster Survey 6
DRG: Diagnosis Related Groups
SSA: Sub Saharan Africa
CAP: Catchment Area Population
MoFEA: Ministry of Finance and Economic Affairs

## References

[1] Cometto G, Buchan J, Dussault G (2020). Developing the health workforce for universal health coverage. Bull World Health Organ.

[2] World Health Organisation (2016). Health workforce requirements for universal health coverage and sustainable development goals.

[3] Reid M, Gupta R, Roberts G, Goosby E, Wesson P (2020). Achieving Universal Health Coverage (UHC): dominance analysis across 183 countries highlights importance of strengthening health workforce. PLoS One.

[4] World Health Organization (2016). Global strategy on human resources for health workforce 2030.

[5] Pittman P, Chen C, Erikson C, Salsberg E, Luo Q, Vichare A (2021). Health workforce for health equity. Med Care.

[6] World Health Organisation (2017). Strategic purchasing for UHC: unlocking the potential. Global meeting summary and key messages.

[7] Cashin C, Gatome-Munyua A (2022). The strategic health purchasing progress tracking framework: a practical approach to describing, assessing, and improving strategic purchasing for universal health coverage. Health Syst Reform.

[8] World Health Organization (2010). The world health report 2010. Health systems financing: the path to universal coverage.

[9] World Health Organization (2019). Purchasing health services for universal health coverage: how to make it more strategic?.

[10] Kazungu JS, Barasa EW, Obadha M, Chuma J (2018). What characteristics of provider payment mechanisms influence health care providers’ behaviour? A literature review. Int J Health Plann Manage.

[11] World Health Organisation. Provider payment methods and strategic purchasing for UHC. World Health Organisation. Regional Office for South-East Asia; 2017. Available from: https://apps.who.int/iris/handle/10665/258894. Cited 2022 18 February.

[12] Barnum H, Kutzin J, Saxenian H (1995). Incentives and provider payment methods. Int J Health Plann Manage.

[13] Andoh-Adjei F-X, Nsiah-Boateng E, Asante FA, Velden KVD, Spaan EJAM (2019). Provider preference for payment method under a national health insurance scheme: a survey of health insurance-credentialed health care providers in Ghana. PLoS One.

[14] Andoh-Adjei F-X, Van Der Wal R, Nsiah-Boateng E, Asante FA, Van Der Velden K, Spaan E (2018). Does a provider payment method affect membership retention in a health insurance scheme? A mixed method study of Ghana’s capitation payment for primary care. BMC Health Serv Res.

[15] Abualbishr A, Aida Isabel T (2019). Provider payment mechanisms: effective policy tools for achieving universal and sustainable healthcare coverage. Universal health coverage.

[16] Secka K. Nurses and midwives embark on industrial action. Churchills Town: Foroyaa; 2021. https://foroyaa.net/nurses-and-midwives-embark-on-industrial-action/.

[17] Bojang T. Public health workes defiant over strike despite govt’s threat of sanctions. Bakau New Town: The Standard; 2022. https://standard.gm/public-health-workers-defiant-over-strike-despite-govts-threat-of-sanctions/.

[18] Lalchandani K, Gupta A, Srivastava A, Usmanova G, Maadam A, Sood B (2021). Correction to: Role of financial incentives in family planning services in India: a qualitative study. BMC Health Serv Res.

[19] Rudasingwa M, Uwizeye MR (2017). Physicians’ and nurses’ attitudes towards performance-based financial incentives in Burundi: a qualitative study in the province of Gitega. Glob Health Action.

[20] Abduljawad A, Al-Assaf AF (2011). Incentives for better performance in health care. Sultan Qaboos Univ Med J.

[21] Ministry of Health (2019). Step- down training of regional health directorate and health facility staff on the revised quality checklist.

[22] IEG Review Team (2021). Gambia, The - GM-Maternal Child Nutr Hlth Results.

[23] Medhin JG (2016). Results-based financing in The Gambia: innovatively contracting communities and health facilities.

[24] Diaconu K, Falconer J, Verbel A, Fretheim A, Witter S (2021). Paying for performance to improve the delivery of health interventions in low- and middle-income countries. Cochrane Database Syst Rev.

[25] Brock JM, Lange A, Leonard KL (2018). Giving and promising gifts: experimental evidence on reciprocity from the field. J Health Econ.

[26] Flodgren G, Eccles MP, Shepperd S, Scott A, Parmelli E, Beyer FR (2011). An overview of reviews evaluating the effectiveness of financial incentives in changing healthcare professional behaviours and patient outcomes. Cochrane Database Syst Rev.

[27] Gambia Bureau of Statistics (2017). The 2015/16 Gambia integrated household survey vol II- socioeconomic- economic characteristics.

[28] Gambia Bureau of Statistics. The Gambia multiple indicator cluster survey 2018. 2018. Available from: https://mics-surveys-prod.s3.amazonaws.com/MICS6/West%20and%20Central%20Africa/Gambia/2018/Survey%20findings/The%20Gambia%202018%20MICS%20Survey%20Findings%20Report_English.pdf. Cited 2021 24 August.

[29] Gambia Bureau of Statistics, ICF. The Gambia demographic and health survey 2019–20. 2021. Available from: https://www.gbosdata.org/downloads/reports-fact-sheets-76. Cited 2021 10 October.

[30] Njie H, Wangen KR, Chola L, Gopinathan U, Mdala I, Sundby JS, et al. Willingness to pay for a national health insurance scheme in The Gambia: a contingent valuation study. Health Policy Plan. 2023;38(1):61–73. https://academic.oup.com/heapol/article/38/1/61/6775654#381296541. 10.1093/heapol/czac089 PMC9849717 36300926

[31] World Health Organization. Provider payment methods and strategic purchasing for UHC: UHC Technical brief. World Health Organization: Regional Office for South-East Asia. 2017. Available from: http://apps.who.int/iris/bitstream/handle/10665/258894/provider_payment_methods_fr_uhc.pdf?sequence=1. Cited 2022 19 December.

[32] Etiaba E, Onwujekwe O, Honda A, Ibe O, Uzochukwu B, Hanson K (2018). Strategic purchasing for universal health coverage: examining the purchaser-provider relationship within a social health insurance scheme in Nigeria. BMJ Glob Health.

[33] Barasa E, Rogo K, Mwaura N, Chuma J (2018). Kenya National Hospital Insurance Fund Reforms: implications and lessons for universal health coverage. Health Syst Reform.

[34] Obadha M, Chuma J, Kazungu J, Barasa E (2019). Health care purchasing in Kenya: experiences of health care providers with capitation and fee-for-service provider payment mechanisms. Int J Health Plann Manage.

[35] Mohammed S, Souares A, Bermejo JL, Sauerborn R, Dong H (2014). Performance evaluation of a health insurance in Nigeria using optimal resource use: health care providers perspectives. BMC Health Serv Res.

[36] Dowd BE, Laugesen MJ (2020). Fee-for-service payment is not the (main) problem. Health Serv Res.

[37] Ikegami N (2015). Fee-for-service payment - an evil practice that must be stamped out?. Int J Health Policy Manag.

[38] Cashin C. Assessing health provider payment systems: a practical guide for countries working toward universal health coverage. Joint Learning Network for Universal Health Coverage. Washignton, D.C: Joint Learning Network for Universal Health Coverage; 2015. Available from: https://www.jointlearningnetwork.org/wp-content/uploads/2019/11/JLN_ProviderPayment_MainGuide_InteractivePDF.pdf. Cited 2022 21 February.

[39] Lai Y, Fu H, Li L, Yip W (2022). Hospital response to a case-based payment scheme under regional global budget: the case of Guangzhou in China. Soc Sci Med.

[40] Robyn PJ, Bärnighausen T, Souares A, Savadogo G, Bicaba B, Sié A (2012). Health worker preferences for community-based health insurance payment mechanisms: a discrete choice experiment. BMC Health Serv Res.

[41] Barber SL, Lorenzoni L, Ong P (2019). Price setting and price regulation in health care: lessons for advancing Universal Health Coverage.

[42] Nsiah-Boateng E, Asenso-Boadi F, Dsane-Selby L, Andoh-Adjei FX, Otoo N, Akweongo P (2017). Reducing medical claims cost to Ghana’s National Health Insurance scheme: a cross-sectional comparative assessment of the paper- and electronic-based claims reviews. BMC Health Serv Res.

